# Tolterodine Tartrate Proniosomal Gel Transdermal Delivery for Overactive Bladder

**DOI:** 10.3390/pharmaceutics8030027

**Published:** 2016-08-31

**Authors:** Rajan Rajabalaya, Guok Leen, Jestin Chellian, Srikumar Chakravarthi, Sheba R. David

**Affiliations:** 1 PAPRSB Institute of Health Sciences, Universiti Brunei Darussalam, Bandar Seri Begawan BE 1410, Brunei; rajan.rajabalaya@ubd.edu.bn; 2 School of Pharmacy, International Medical University, Bukit Jalil, Kuala Lumpur 57000, Malaysia; guok_leen@hotmail.com (G.L.); pharmjestin@gmail.com (J.C.); 3 School of Medicine, Perdana University, Jalan MAEPS Perdana, Serdang, Selangor 43400, Malaysia; activedoctor@gmail.com

**Keywords:** Proniosome, transdermal, salivary secretion, permeation, cholesterol, overactive bladder

## Abstract

The goal of this study was to formulate and evaluate side effects of transdermal delivery of proniosomal gel compared to oral tolterodine tartrate (TT) for the treatment of overactive bladder (OAB). Proniosomal gels are surfactants, lipids and soy lecithin, prepared by coacervation phase separation. Formulations were analyzed for drug entrapment efficiency (EE), vesicle size, surface morphology, attenuated total reflectance Fourier transform infrared (ATR-FTIR) spectroscopy, in vitro skin permeation, and in vivo effects. The EE was 44.87%–91.68% and vesicle size was 253–845 nm for Span formulations and morphology showed a loose structure. The stability and skin irritancy test were also carried out for the optimized formulations. Span formulations with cholesterol-containing formulation S1 and glyceryl distearate as well as lecithin containing S3 formulation showed higher cumulative percent of permeation such as 42% and 35%, respectively. In the in vivo salivary secretion model, S1 proniosomal gel had faster recovery, less cholinergic side effect on the salivary gland compared with that of oral TT. Histologically, bladder of rats treated with the proniosomal gel formulation S1 showed morphological improvements greater than those treated with S3. This study demonstrates the potential of proniosomal vesicles for transdermal delivery of TT to treat OAB.

## 1. Introduction

Overactive bladder (OAB) syndrome was defined as “urgency, with or without urge incontinence, usually with frequency and nocturia,” by the International Continence Society [1]. This leads to a decrease in quality of life. A prevalence of 22 and 12 percent was reported in Spain and France, respectively. In Asia, the reported prevalence was even greater, 30%, and increased with age [2]. The most common type of urinary incontinence among geriatric patients is urge incontinence, as bladder contraction is predominantly stimulated by parasympathetic innervation, anticholinergic agents are used for treatment and are the first-line drugs for women with OAB [3].

Antimuscarinics most currently used to treat OAB are oral oxybutynin, tolterodine, and trospium, which have associated side effects such as dry mouth, constipation, headache, and blurred vision, often leading to discontinuation of therapy [4,5]. Tolterodine tartrate (TT) is more desirable than oxybutynin because it has greater bladder selectivity and lower salivary affinity, making it better tolerated [6]. However, its anticholinergic side effects, especially dry mouth, have remained a common reason for therapy discontinuation [7]. It was reported that 30% of oral TT treated patients experienced mild dry mouth, compared with 8% of placebo-treated patients, demonstrating the poor tolerability of an oral TT formulation [8]. There are very few studies on transdermal drug delivery (TDD) of tolterodine because no transdermal formulation is available in the market. A TT transdermal patch was described with a pharmacokinetic profile comparable to that of a conventional oral formulation [9]. A micro-emulsion containing tolterodine tartrate showed sustained activity for over 72 h after application because of the continuous replenishment of the drug from the vesicular system into the systemic circulation [10]. A transdermal formulation had a lower incidence of adverse effects such as dry mouth and constipation [9]

Vesicular systems, especially proniosomes, self-assembled uni- or multilamellar bilayered spheroidal structures covered with non-ionic surfactants, are becoming increasingly popular [11,12,13]. Their advantages include greater physical stability than niosomes, higher drug loading capacity than liposomes, and suitability for application on dry skin. Therefore, our study focused on development and evaluation of proniosomal gels incorporated with the antimuscarinic TT, including addressing their potential side effects and efficacy in OAB treatment.

## 2. Materials and Methods

TT was provided by Aurobindo Pharmaceuticals (Hyderabad, India). Span^®^ surfactants, cholesterol, soy lecithin, glycerine, acetic acid, pilocarpine and neutral buffered 10% formalin solution were from Sigma–Aldrich (St. Louis, MO, USA). Glyceryl distearate (Gattefosse, Saint-Priest, France), Wecobee^®^ M, a triglyceride derived from fully hardened palm kernel oil (conforms USP/NF monograph) from Stepan (Northfield, IL, USA) and isopropyl alcohol (IPA) from Merck (Darmstadt, Germany).

### 2.1. Preparation of Proniosomal Gels

Proniosomal gels were prepared by coacervation phase separation method [14]. Surfactants, lipids and drug, together with 0.5 mL isopropanol, were placed in a glass vial which was capped and warmed in a water bath at 40–60 °C for approximately 20 min until the mixture was dissolved. Then 0.5 mL 0.1% glycerine was added and the vial warmed again until a clear solution formed. A proniosomal gel was obtained upon cooling of the solution to room temperature, and was kept in the dark until further evaluation. Compositions of proniosomal gels are shown in Table 1.

### 2.2. Characterization of Proniosomal Gels

#### 2.2.1. Entrapment Efficiency (EE)

Proniosomal gel (100 mg) was dispersed in 10 mL phosphate buffer saline, pH 7.4 (PBS). The vesicles were pelleted by centrifugation at 12,000× *g* at 5 °C for 30 min (5810R; Eppendorf, Hamburg, Germany). The supernatant layer was collected to measure free drug by high-performance liquid chromatography (HPLC) [15]. The percentage EE (Table 1) was calculated using the following equation.

(1) $$\%\;\text{Entrapment}\;\text{efficiency}=\frac{\text{Total}\;\text{drug}\;\text{content}-\text{drug}\;\text{content}\;\text{in}\;\text{supernatant}}{\text{Total}\;\text{drug}\;\text{content}\;}\times 100$$

#### 2.2.2. Vesicle Size

The particle size, polydispersity index (PDI), of pronisomes were measured using a Malvern Zetasizer (NanoZS90, Worcestershire, UK) at 25 °C and a 90° scattering angle. A 1:100 dilution of the sample was made using double distilled water before the measurements to get 50–200 optimum kilo counts per second (Kcps) for measurements. Results are shown in Table 1.

#### 2.2.3. Surface Morphology

A thin layer of proniosomal gel formulation was spread on a glass slide, a drop of distilled water was added, and the slide was examined under an optical microscope to assess whether the sample formed vesicles or had precipitated drug. With confirmation of vesicle formation, the same procedure was followed to place the gel sample on a holding disk for analysis inside an Environmental Scanning Electron Microscope (eSEM: Quanta 450 FEG; FEI, Hillsboro, OR, USA) chamber. Water within the sample was allowed to evaporate before examination and photographs were taken at a 10 kV accelerating voltage [16].

#### 2.2.4. pH Measurement

The pH of the proniosomal gel were determined by a digital pH meter (Mettler Toledo, Greifensee, Switzerland). 1 g of gel was dissolved in 20 mL of distilled water and the electrode was then dipped into gel formulation and constant reading was noted. The measurements of pH of each formulation were replicated three times.

### 2.3. Stability Study

Optimized formulations S1 and S3 were stored at three different temperatures: refrigeration (2–8 °C), room (25 ± 2 °C) and oven (45 ± 2 °C) [12]. After three months, EE was determined (Table 2), as described previously under EE determination.

### 2.4. Attenuated Total Reflectance-Fourier Transform Infrared (ATR-FTIR) Spectroscopy

The spectra of gel formulations and individual components (drug, surfactants and lipids) were obtained on a Magma-IR™ Spectrometer 750 (Nicolet Instruments, Madison, WI, USA) equipped with a Golden Gate™ Single Reflection Diamond Attenuated Total Reflectance (ATR) system. The spectra were determined from an average of 20 scans with a resolution of 4 cm^−1^ and in the frequency range of 4000–650 cm^−1^. Spectra are presented in Figure 3 and Figure 4.

### 2.5. In Vitro Permeation Studies

Vertical Franz Diffusion Cells (PermeGear, Hellertown, PA, USA), with an area of 1.7671 cm^2^ donor compartment, were used for in vitro skin permeation studies. An aliquot (0.1 gm) of proniosomal gel was applied to a Strat-M™ membrane (Merck Millipore, Billerica, MA, USA), an alternative to animal skin. The receptor cell (12 mL capacity) was jacketed to maintain 32 °C and had a magnetic bead for uniform stirring. The sample (1 mL) from the receptor compartment was collected every hour for 8 h and an equal volume of fresh phosphate buffer saline, pH 7.4, was added immediately [16,17]. The amount of TT, from the proniosomal gel, in each sample was analyzed by reverse-phase HPLC eluted at 1 mL/min with a mobile phase of methanol:20 mM pH 3.0 acetate buffer (65:35, *v*/*v*), measured at 281 nm with a ultraviolet (UV) detector [15]. Likewise for in vitro permeation studies, freshly excised rat skin was used under same conditions.

### 2.6. In Vivo Studies

#### 2.6.1. Skin Irritancy Test

The skin irritancy test was performed with slight modifications to the mentioned method on male albino rats (150–180 g) to determine localized skin reaction [18]. Four groups of animals were used: the first group received the irritant, 0.8% (*v*/*v*) aqueous formalin solution, the second group was the control (untreated), and the third group was administered the S1 gel, while the fourth group received S3 gel, to evaluate skin irritation. The optimized gel (1 g) or formalin solution (1 mL) was applied to the posterior side of the shaved dorsal region at the back of rats (5 cm^2^ area) once a day for three consecutive days. The observed localized reactions, edema and erythema, daily for three days are as shown in Table 4.

#### 2.6.2. Acetic Acid (ACA)-Induced Bladder Hyperactivity

Acetic Acid (ACA) solution (0.75%) was administered to unconscious rats by a urinary catheter. In this model, chemical irritation caused by ACA produces acute inflammation of the urinary bladder and, consequently, facilitates micturition in rats [19]. Bladder inflammation was evaluated histopathologically. All in vivo experiment procedures and animal care permission letter (B0109_Res182012) for approval of animal experimentation by International Medical University (IMU) Research Ethical Committee.

#### 2.6.3. Salivary Secretion

Measurement of salivary secretion study was performed after the rats were anesthetized with diethyl ether [3]. We used three to five cotton balls to absorb the saliva for 10 min, and these were weighed immediately with a balance prior to any moisture loss. The treatment (oral/gel) was given at 0 h and the pilocarpine-induced salivary secretions were assessed at 2, 4, 12, and 24 h [3].

#### 2.6.4. Histopathology

The urinary bladder was removed from each rat and immediately immersed in 10% neutral buffered formalin solution. After processing, embedding, and sectioning, 5-mm-thick tissue sections were stained with hematoxylin followed by eosin and observed under a light microscope.

### 2.7. Statistical Analyses

Statistical analysis of data was performed with SPSS v8.0 (IBM Corp., Armonk, NY, USA). Analysis of variance and the paired *t*-test were applied. *p* < 0.05 was considered significant. Values are expressed as means ± standard deviation.

## 3. Results

### 3.1. EE, Vesicle Size, and Stability Study

The different surfactants and lipids used to prepare TT proniosomal gels are shown in Table 1, along with the EE and vesicle size of each formulation. All Span series formulations showed EE more than 80%, except for S5 and S6 formulations. The Span series formulations vesicle sizes ranges from 253 to 845 nm. Among gel formulations, S1, S2, and S3 had smaller vesicle sizes, with added cholesterol (50 mg), EE was high in the S1 formulation (91.68%), but with even more cholesterol (100 mg), EE became lower (86.45%) in the S2 formulation. Addition of another lipid, Wecobee^®^ M, in S4, led to a marked decrease in EE (*p* < 0.05), compared with that of S3. S1 had a significantly higher EE than S2 (*p* < 0.05), the latter containing twice the amount of cholesterol. Thus, the optimum Span: cholesterol ratio was 20:1. S2 and S3 had the same composition of surfactants but different lipids, cholesterol, and glyceryl distearate, respectively. However, their EE values were not significantly different (*p* > 0.05). The pH of the formulations is suitable for skin (Table 1).

Differences in EE, for stability studies, which were determined based on drug content are presented in Table 2. The formulation S1 had higher drug content in all the temperatures compared to S3. The drug content of S3 is in the range of 80%–84% after three months of the study.

### 3.2. Surface Morphology

**Figure 1:**
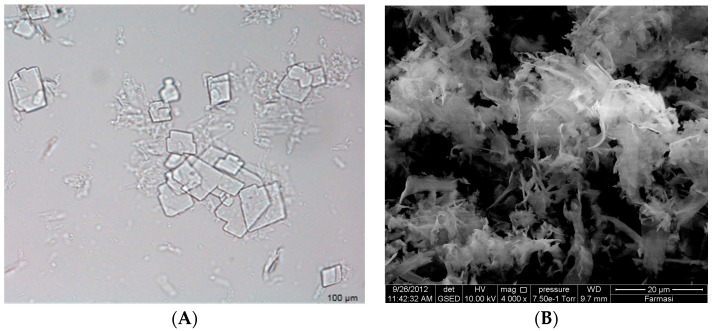
Insoluble drug crystals of S1 formulation (40 mg) observed under optical microscope (**A**) and Environmental Scanning Electron Microscope (eSEM) (**B**).

**Figure 2:**
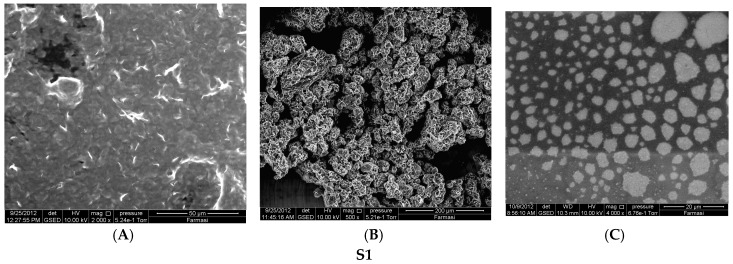

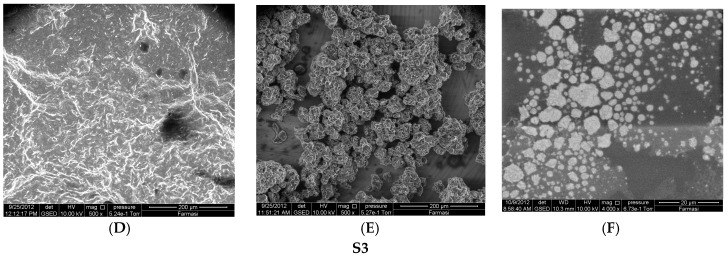
Environmental Scanning Electron Microscope (eSEM) images for the proniosome formulations S1 (**A**–**C**); S3 (**D**–**F**) without hydration, less hydration (2 mL), and more hydration (5 mL), respectively.

Increasing the drug load from 20 to 40 mg in the S1 formulation resulted in a non-significant decreasing trend of EE from 91.68% to 89.45% (*p* > 0.05). Insoluble drug crystals were observed with higher drug (40 mg) as seen in Figure 1A,B. However, 20 mg TT formulations did not form crystals (Figure 2B,C,E,F) neither in less hydration (2 mL of double distilled water) nor in higher hydration (5 mL of double distilled water), and this concentration was used for further studies. The proniosome formulations examined before hydration, less hydration and more hydration by eSEM are shown for the formulations S1 (Figure 2A–C) and S3 (Figure 2D–F), respectively. S1 and S3 (Figure 2C,F) showed a loose structure probably because of the absence of Wecobee^®^ M. From the eSEM analysis of S1, the micelle surfaces appeared to be coated with drug [11].

### 3.3. ATR-FTIR Analysis

**Figure 3:**
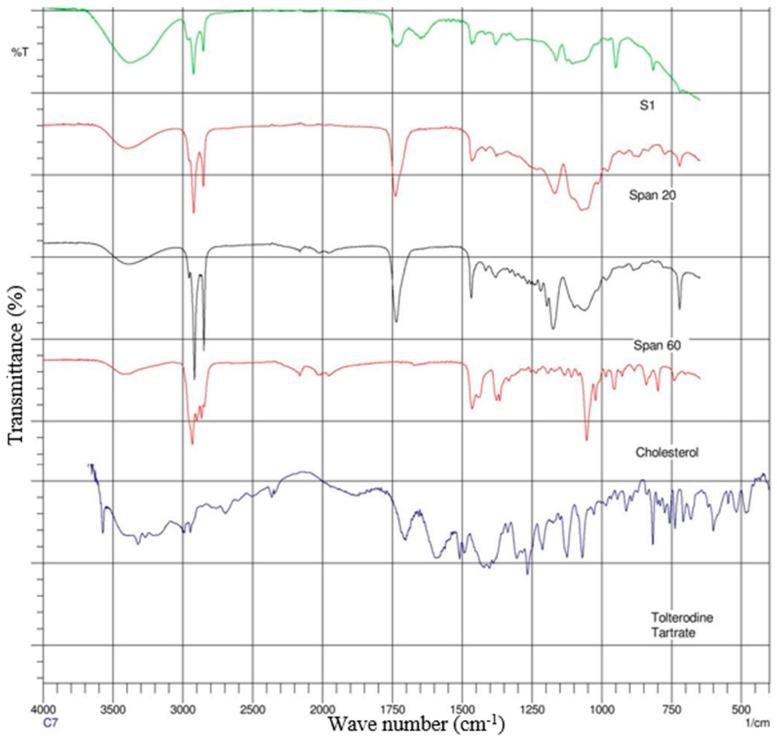
Attenuated total reflectance Fourier transform infrared (ATR-FTIR) spectra of formulation (S1) and its individual components.

**Figure 4:**
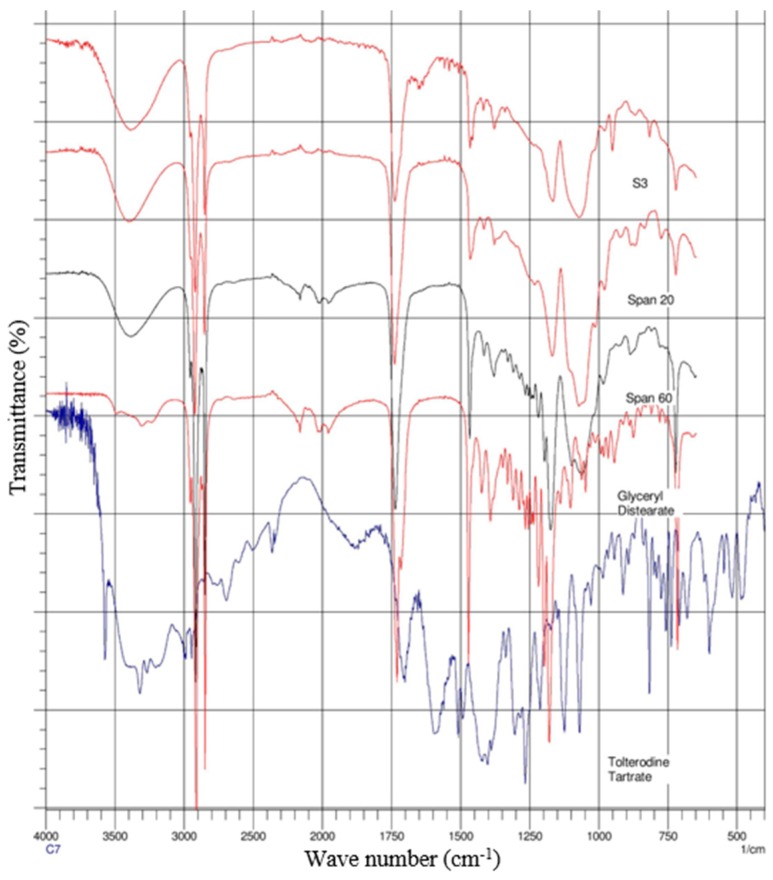
ATR-FTIR spectra of formulation (S3) and its individual components.

ATR-FTIR spectroscopy was used to scan individual excipients and the gels S1 and S3 (Figure 3 and Figure 4), at a resolution of 4 cm^−1^ in the frequency range 650–4000 cm^−1^, to detect any spectral shifts occurring in components within proniosomal gels. FTIR spectra of TT, cholesterol, Span 20, Span 60, and TT; cholesterol, Span 20, Span 60, and glyceryl distearate with proniosomal gels S1 and S3 are shown in Figure 3 and Figure 4, respectively.

### 3.4. In Vitro Permeation Studies

**Figure 5:**
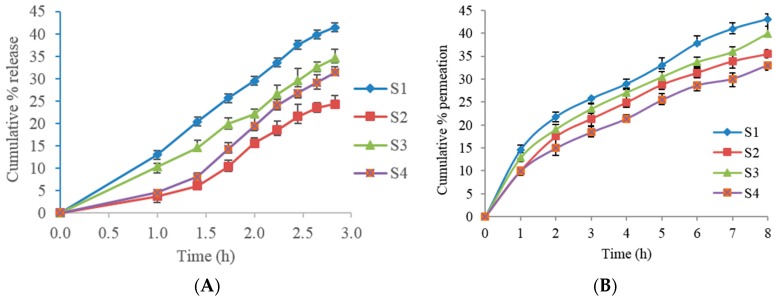
Cumulative percent permeation over 8 h following application of proniosome gels, S1–S4. (**A**) With Strat-M™ membrane; (**B**) with freshly excised albino rat skin.

In vitro permeation studies were performed for S1 to S4 formulations. The formulations S5 and S6 were excluded due to lower entrapment efficiency (Table 1) with 56.8% and 44.9% respectively, when compared to other formulations. Two different membranes, rat skin as well as Strat-M™ membrane, were utilized for these experiments. The cumulative drug permeation percentage versus time for several Span formulations are shown in Figure 5A (with Strat-M™ membrane) and 5B (freshly excised albino rat skin). In the S1 formulation containing 50 mg cholesterol, the percent of permeation was slightly higher in rat skin compared to Strat-M™ membrane with, 43.03 and 41.43, respectively, at the 8 h. While further increasing the cholesterol in S2 formulation to 100 mg of cholesterol, the percent of permeation markedly decreased when compared to S1 formulation, at the 8 h, in both membranes. The cumulative percentage permeation from S3 formulation was 39.93 in rat skin. It was observed that S4 formulation had the least percentage cumulative amount of TT with less than 35%, in both membranes. The in vitro release data from Strat-M™ membrane was fitted to various kinetic models to determine the release mechanism of TT from the proniosomal gel (Table 3).

### 3.5. In Vivo Studies

#### 3.5.1. Skin Irritancy Test

The skin irritancy test was conducted to determine the safety of proniosomal gels (S1 and S3). The results are shown in Table 4. According to classical in vivo skin irritancy assay test by Lehman [20], non-irritants to skin have a primary irritancy index (PII) of less than 2. Thus, S1 and S3 were considered to be non-irritants to the skin.

#### 3.5.2. Salivary Secretion

**Figure 6:**
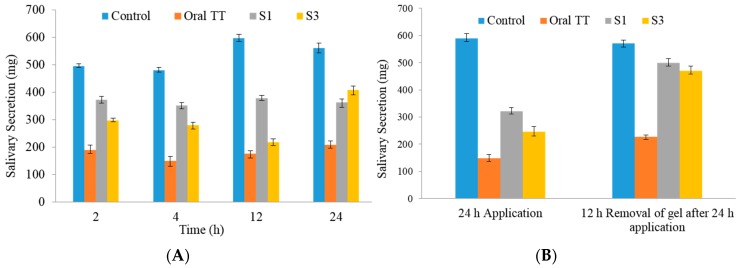
Salivary secretion in rats with various formulations over 24 h (**A**) control (no tolterodine tartrate (TT) treatment) compared with oral, S1, and S3 TT formulations; (**B**) Recovery of pilocarpine-induced salivary secretion after removal of gel formulation. *n* = 6.

Based on EE and in vitro permeation, S1 and S3 formulations were selected for testing in vivo. Salivary secretion at various times is shown in Figure 6. The amount of saliva secreted was significantly decreased in rats treated with oral and transdermal gel TT formulations, as compared with in untreated controls (*p* < 0.05). With oral TT the total saliva collected (*p* < 0.05) was less at all time points (189.4 ± 4.04, 149.4 ± 3.68, 176.3 ± 2.99, and 208.4 ± 2.16 mg at 2, 4, 12 and 24 h, respectively, *n* = 6) (Figure 6A), compared with both S1 and S3 gel formulations. From Figure 6B, it is clear that the total saliva secreted (at 24 h) in groups treated with S1 (494.97 ± 9.32 mg, *n* = 6) or S3 (479.23 ± 10.47 mg, *n* = 6) was significantly more than in the rats receiving the oral formulation (208.4 ± 2.16 mg, *n* = 6). The amounts of total saliva secreted in the first 10 min in control, oral TT treated, S1 treated, and S3 treated groups were similar after pilocarpine stimulation (Figure 6A).

The saliva secretion in rats receiving oral or transdermal TT was significantly lower than those in controls (*p* < 0.05). However, the oral TT resulted in a more marked decrease in total saliva collected (*p* < 0.05) at all time points (Figure 6A), than in rats receiving the S1 and S3 gel formulations. S1 formulation has almost uniform salivary secretion from 2 to 24 h whereas S3 has fluctuations. The salivary secretion for S3 is decreasing from 2 to 12 h, while it increased significantly at 24 h. Transdermal application of S1 and S3 gels markedly decreased pilocarpine-induced salivation (Figure 6A). Nonetheless, the respective weights (371.83, 351.84, 378.84, and 326.36 mg) of total saliva secreted at 2, 4, 12, and 24 h were significantly higher (*p* < 0.05) than those after oral administration of TT. However at 24 h the weights of saliva collected were markedly higher for S3 compared with S1, indicating that gel administration for the latter is required after 12 h. Compared with the S1 formulation, S3 showed less drug permeation in vitro (Figure 5). As shown in Figure 6B, pilocarpine-induced salivation recovered 12 h after transdermal TT was removed.

#### 3.5.3. Histopathology in Acetic Acid Induced Bladder Inflammation

**Figure 7:**
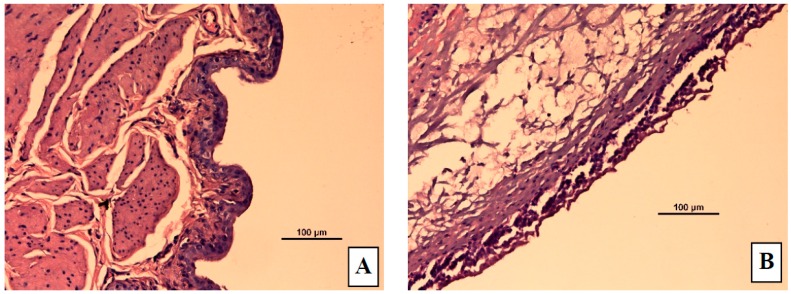

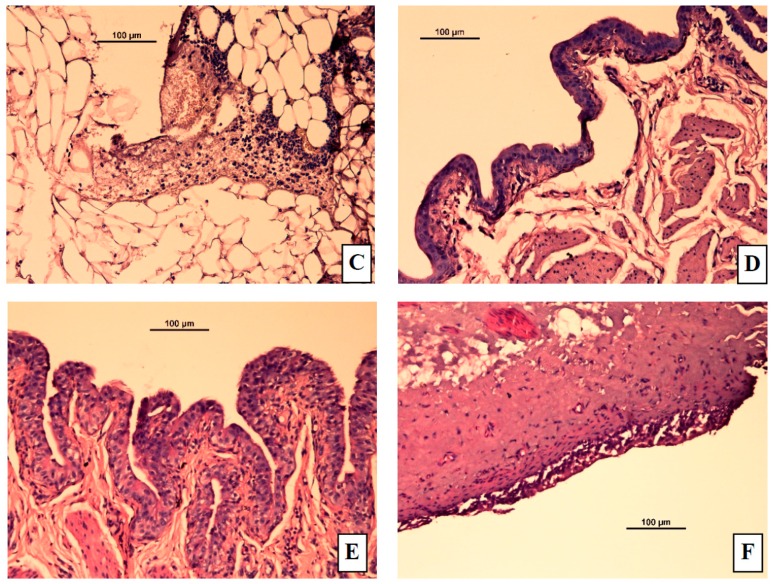
Representative micrographs from rat bladders immediately after micturition studies. (**A**) Control (normal) group (**B**) Acetic acid treated group showing edema (**C**) Acetic acid treated group showing necrosis and ulceration (**D**) Acetic acid treated group given tolterodine by oral (**E**), S1 gel, or (**F**) S3 gel formulations. In the S1 gel treated group, the transitional epithelium regenerated quickly and in the S3 gel treated group, the bladder structure is similar to that in S1 but with more mild mucosa ulceration and scattered inflammation.

The control group showed a normal urinary bladder with multiple layers of epithelial cells. The transitional epithelium was resting on a submucosal layer comprising fibrous connective tissue, with an underlying layer of irregular muscle fibers (Figure 7A). In the ACA-induced group (Figure 7B,C), necrosis of the transitional epithelium and focal areas of discontinuity and ulceration of the mucosa were observed. Submucosal and muscle layers showed inflammatory infiltrates composed predominantly of neutrophils and scattered lymphocytes. Histology of bladders from TT, S1, and S3 treated rats are shown in Figure 7D–F, respectively. S1 and S3 treated groups showed highly regenerative surfaces on the transitional epithelium.

## 4. Discussion

### 4.1. Entrapment Efficiency, Vesicle Size, and Stability Study

In this study, the formulation with the most efficient entrapment of TT was obtained with a combination of Span 20 and 60 at a 1:1 ratio, possibly because Span 60 is at a solid state at room temperature and has a higher phase transition temperature (Tc) [21,22]. TT is a hydrophobic drug and will be encapsulated in the lipid bilayer of the vesicle. Moreover, Span series surfactants have low Hydrophilic Lipophilic Balance (HLB) values therefore, they produced formulations with higher EE values, consistent with previous reports [23,24]. The ability of sorbitan monostearate (Span 60) to form vesicles depends on its structure, critical packing parameter (CPP), hydrophilic–lipophilic balance, and presence of cholesterol. The relatively large hydrophobic moieties in Span 60 may lead to intercalation of TT into the bilayers leading to an increased affinity among the nonpolar portions of the membrane [25]. Moreover, Span 60 has higher entrapment efficiency due higher phase transition temperature, thus showing bilayer flexibility and capacity of reorganization during hydration [26].

Cholesterol interacts with surfactants, influencing their physical properties and vesicle structures by modulating cohesion and mechanical strength of bilayers, causing an increased EE [27]. Addition of cholesterol can decrease the gel–liquid transition temperature of vesicles thus making them more rigid and less permeable to drug leakage, which would improve EE [11]. Addition of another lipid, Wecobee^®^ M, to Span formulations led to a marked decrease in EE probably because of an increase in bilayer hydrophobicity and larger vesicle size, both contributing to a lower EE [28]. Addition of cholesterol (50 mg) increased EE, however, further increase of cholesterol (100 mg) did not increase it further; these results are consistent with previous reports [11,29]. This is probably due to the competition between cholesterol and drug for packing space within the bilayer [30].

Glyceryl distearate plays the same role as cholesterol in influencing this parameter. Formulation with Span 40 have a lower EE than Span 60, perhaps because the shorter alkyl chains and lower phase transition temperature (Tc) of the former would form less ordered bilayers, decreasing stability and, therefore, EE [11,21]. EE was described as being proportional to the chain length of surfactants [29,31].

At higher temperature, there is a higher leakage from proniosome formulation vesicles, whereas good stability was observed at lower temperatures (2–8 °C). This may have been the result of vesicle loosening and higher fluidity of lipid bilayers at higher temperature [12]. Cholesterol maintains EE, by modulating cohesion and mechanical strength of lipid bilayers leading to stable vesicle structures [32], whereas formulations without cholesterol showed slight decrease of EE at higher temperatures [33]. Polydispersity index showed that the formulations were uniform.

### 4.2. Surface Morphology

Niosomes are formed by increasing hydration of proniosomes in aqueous media. Span formulations containing cholesterol and lecithin had loose structures probably because of the combined absence of Wecobee^®^ M and glyceryl distearate; While the absence of Wecobee^®^ M alone, produced less loose structures. Also because of the higher hydrophobicity of Span, spherical vesicles are not formed with the addition of water. Moreover, higher hydrophobic surfactants become too bulky and do not fit into a spherical shape when contacted with aqueous media. In addition, the packing of vesicles appeared like a hexagonal system with more hydration. The hydrophilic TT is attracted to the hydrophilic region on the outer surface of micelles, which can contribute to an increased EE, leading to larger surface areas enabling higher drug loading.

### 4.3. ATR-FTIR Spectroscopy

Interactions between cholesterol and Span 60 in the bilayer of niosomes involve hydrogen bonding [32], leading to aggregation and bilayered vesicles during niosome formation. The spectrums of the TT, cholesterol, Span 20, Span 60 with S1 gel are displayed in Figure 3 while TT, cholesterol, Span 20, Span 60 and glyceryl distearate with S3 gel mixture peaks are showed in Figure 4. The spectrums of the drug and combined mixtures revealed a band due to –OH stretching at 3457.07 cm^−1^; there were bands recorded for –C–H stretching (aromatic) at 3040.10 and 3012.07 cm^−1^; while –CH_3_ stretching (aliphatic) resulted in 2685.15 and 2934.90 cm^−1^; furthermore C=C stretching (aromatic) resulted 1604 and 1454 cm^−1^ bands. The respective shifts from surfactants span 20 and 60 at 1750, 2950 and 1450 cm^−1^, 2900 cm^−1^ were also found in the gels.

Figure 4 depicts the S3 gel formulation with glyceryl distearate lipid which produced its peaks. This has overwhelmed the other peaks with slight noise in the spectrum. However, the compatibility between surfactants and drug was clear from the ATR-FTIR study, the peaks corresponding to either surfactants or drug did not show any significant shifts in the spectrum.

### 4.4. In Vitro Permeation Studies

In vitro permeation studies should be performed in a non-occlusive environment to better simulate the topical application of proniosomal gels [11]. Span based proniosomes produced slight bursts of drug initially in rat skin compared to Strat-M™ membrane. This is possibly indicating that firstly, the partition into the stratum corneum (SC) from the formulation vehicle, isopropanol, and secondly, diffusion into the SC and partitioning from the SC into rat skin than Strat-M™ membrane. The solubility of the drug in the vehicle will influence both the drug concentration gradient in the solution and partition coefficient between the vehicle and the membrane [34]. Thirdly, the presence of cholesterol in a proniosome can influence the permeation of TT because of the interaction of the niosomes with rat membranes. Thus, drug permeability is elevated by increased membrane fusion processes and elasticity, as well as changes in size and shape [27].

Concerning surfactant and cholesterol desorption of the drug from the surface of the vesicles. The following slower, permeation phase might have indicated drug diffusion from swollen bilayers in contact with water on the buffer saline solution [35]. In all permeation profiles, the cumulative permeation percentage increased over at least 8 h and obeyed concentration dependent first order kinetics with the rat skin. The release percentages of both, Strat-M™ and rat skin, membranes were observed to have similar results, hence Strat-M™ membrane is a suitable alternative for drug release studies. The reason for the similar results may be due to the moistening by PBS, converting proniosomes into niosomes. This conversion can lead to changes in consistency and fluidity, resulting in flexible vesicles that enable increased drug diffusion [36]. Span 60, with a higher Tc and longer alkyl chain than Span 40, will form a more ordered gel leading to less leaky formulation [11,21]. Shorter alkyl chains also increase drug permeation as compared to longer alkyl chains [23,37,38].

In the Span series, glyceryl distearate or cholesterol addition led to similar permeation percentages (*p* > 0.05), suggesting that these lipids play similar roles in promoting drug permeation. The formulation with the lower amount of cholesterol had better drug permeation, unlike the formulation with the higher amount. Perhaps, this might be attributed to its higher amount of cholesterol which may decrease the leakage and permeability of TT from the proniosomal vesicles leading to lesser permeation. Glyceryl distearate may disrupt the lipids in the stratum corneum allowing vesicle penetration [27]. Glyceryl distearate containing formulations might enhance the drug partitioning in the skin resulting in higher drug permeation, however the permeation percentages were lower compare to cholesterol based formulations.

### 4.5. In Vivo Studies

#### 4.5.1. Skin Irritancy Test

It is essential to have good skin compatibility without irritation for transdermal delivery formulation as it stays in the skin longer. Thus the obtained results clearly signify that the proniosomal gel achieved the objectives of controlled drug release. The Span surfactants of the proniosomal gels had longer alkyl chain length leading to gradual release of the drug into the skin layer over longer period of time. This was helpful to reduce the irritation and toxicity of drug [18].

#### 4.5.2. Salivary Secretion

In the salivary secretion model, orally administered TT was reported to bind more strongly to the salivary gland, reach the systemic circulation and act at the cholinergic receptor more than transdermally administered TT [39]. Total saliva collected was decreased in the hours after administration of proniosome gel formulations, indicating that transdermal TT was exerting effects on salivary glands. These effects could be correlated with results of the skin permeation study, in which the percentage of permeation increased throughout the experiment. In addition, pilocarpine-induced salivation recovered quickly, almost attaining normal salivation levels, within 12 h of gel removal. M2 receptor subtypes are predominant in urinary bladder, whereas M3 subtype has major control on the contraction modulation over M2 subtype in bladder [40], however, in various pathological states, M2 receptors modulate detrusor contraction by several mechanisms [41,42]. Additionally, many studies have clearly established the advantage of transdermal over oral route for tolterodine for OAB [10]. These observations indicated that the TT proniosomal gel had favorable properties, causing less dry mouth and allowing faster recovery of salivation, compared with the oral formulation. Moreover, the binding of oral tolterodine to muscarinic receptor had slower onset and longer duration [19].

#### 4.5.3. Histopathology in the Bladder Inflammation Model

From the histopathological analysis, there was a clear difference between rats in the control group, with a normal intact urinary bladder, and those in the acetic acid treated group, which showed transitional cell necrosis as well as mucosal ulceration and inflammatory infiltration indicating acute cystitis. The inflammatory infiltrates consisting of neutrophils and scattered lymphocytes, in submucosal and muscle layers, suggest an acute form of inflammation in the bladder tissue that extended throughout its thickness. The bladders in rats receiving oral TT showed nearly normal bladder structures, indicating complete bladder healing. Regenerative surface is one of the indications of the treatment modality efficacy.

However, the mucosal surfaces in bladders of transdermal TT treated rats had numerous papillary folds clustered with transitional cells. Furthermore, the submucosa in these bladders showed evidence of fibrosis and blood vessels, suggesting healing scar tissue. S3 formulation appeared to induce rapid transitional epithelium regeneration with mild mucosal ulceration; however, complete regeneration may be possible with long term treatment.

## 5. Conclusions

Our findings suggest that, among the formulations studied, the S1 gel had the most effective combination of surfactant, cholesterol, and aqueous phase. This formulation had the highest EE and drug permeation properties. In the in vivo studies, rats administered with S1 had higher salivary secretion, compared with oral and S3 formulations; moreover, we observed faster regeneration of epithelium in the bladder for S1 compared with the S3 formulation. Thus, our study has established that transdermal TT formulations can have efficacies comparable with those of oral formulations for OAB and, also, cause less dry mouth effects. These results can serve as a foundation for future studies on the potential of proniosomal gels for transdermal TT delivery.

## Figures and Tables

**Table 1:** Compositions, entrapment efficiencies (EE%) vesicle sizes with polydispersity index and pH of various proniosomal formulations.

Code	Surfactant Type	Ratio (mg)	Wecobee (mg)	Cholesterol (mg)	Glyceryl Distearate (mg)	Lecithin (mg)	Entrapment (%)	Vesicle Size (nm)	Polydispersity Index	pH
S1	S20:S60	500:500	-	50	-	100	91.7 ± 1.0	145.1 ± 5.3	0.4 ± 0.1	6.61 ± 0.5
S2	S20:S60	500:500	-	100	-	100	86.5 ± 1.1	178 ± 7.9	0.5 ± 0.2	6.80 ± 0.7
S3	S20:S60	500:500	-	-	100	100	88.4 ± 0.4	170.3 ± 11.4	0.5 ± 0.1	6.19 ± 0.9
S4	S20:S60	500:500	100	-	100	100	84.2 ± 0.7	447.6 ± 20.0	0.7 ± 0.2	6.58 ± 0.5
S5	S20:S40	500:500	100	-	100	100	56.8 ± 1.3	253.2 ± 23.4	0.7 ± 0.0	5.73 ± 0.9
S6	S40:S60	500:500	-	-	100	100	44.9 ± 3.7	348.2 ± 31.5	0.6 ± 0.2	5.89 ± 1.1

**Table 2:** Change in entrapment efficiency after storage for 3 months (Stability studies data).

Gel Code	Period	Drug Content (%)		
		5 ± 2 °C	25 ± 0.5 °C	45 ± 0.5 °C
S1	Initial	91.68 ± 0.99	91.68 ± 0.99	91.68 ± 0.99
	After 3 months	89.18 ± 2.06	88.02 ± 1.89	86.12 ± 2.08
S3	Initial	88.36 ± 0.41	88.36 ± 0.41	88.36 ± 0.41
	After 3 months	85.98 ± 0.81	82.36 ± 1.07	80.08 ± 1.27

**Table 3:** Release kinetics data treatment for proniosomal gel formulations.

Formulation Code	Zero Order		First Order		Higuchi		Korsmeyer-Peppas Model	
	*r*^2^	*K_0_ (h*^−1^*)*	*r*^2^	*K_1_ (h*^−1^*)*	*r*^2^	*K_H_*	*r*^2^	*n*
S1	0.998	2.678	0.987	0.081	0.991	17.257	0.995	0.81
S2	0.985	3.147	0.923	0.248	0.985	20.341	0.962	0.51
S3	0.997	3.067	0.989	0.173	0.990	19.653	0.989	0.65
S4	0.983	3.252	0.961	0.146	0.984	22.347	0.973	0.59

**Table 4:** Skin irritancy test, Visual observation values were expressed as Mean ± SD, *n* = 6; S1 proniosomal gel; S3 proniosomal gel; Control = untreated rats. Erythema scale: 0, none; 1, slight; 2, well defined; 3, moderate; and 4, scar formation. Edema scale: 0, none; 1, slight; 2, well defined; 3, moderate; and 4, severe.

Rats	Control		Formalin Solution		S1 Gel		S3 Gel	
	Erythema	Edema	Erythema	Edema	Erythema	Edema	Erythema	Edema
1	0.0	0.0	4	2	0.0	0.0	0.5	0.0
2	0.0	0.0	4	1	0.5	0.0	1	0.0
3	0.0	0.0	3	3	1	0.0	1	0.0
4	0.0	0.0	3	3	1	0.0	0.5	0.0
5	0.0	0.0	3	3	1	0.0	1	0.0
6	0.0	0.0	4	3	1	0.0	1	0.0
Mean	0.0	0.0	3.5	2.5	0.75	0.0	0.83	0.0
SD	0.0	0.0	0.75	0.53	0.32	0.0	0.28	0.0
PII	0.0 ± 0.0		6.0 ± 1.36		0.75 ± 0.25		0.83 ± 0.31	

PII—Primary irritancy index (*n* = 6).
